# Supplementary material for: Efficacy and Safety of Adding Ribavirin to Sofosbuvir-Based Direct-Acting Antivirals (DAAs) in Re-Treating Non-Genotype 1 Hepatitis C—A Systematic Review and Meta-Analysis
Source: Diseases. 2025 Apr 29;13(5):138. doi: 10.3390/diseases13050138 (PMC12110649; doi:10.3390/diseases13050138)
Supplement: Supplementary file 1 [file diseases-13-00138-s001.zip › S2- search strategy.pdf]

search strategy

PubMed search

Search #1 Search terms for DAAs (n= 281 417)

"sofosbuvir" OR "sovaldi" OR "simeprevir" OR "olysio" OR "daclatasvir" OR "daklinza" OR "ledipasvir" OR "harvoni" OR "elbasvir" OR "grazoprevir" OR "zepatier" OR "velpatasvir" OR "epclusa" OR "ombitasvir" OR "paritaprevir" OR "dasabuvir" OR "viekira pak" OR voxilaprevir OR ritonavir OR 3D OR glecaprevir OR pibrentasvir OR mavyret OR "direct-acting agents" OR “direct acting antiviral” OR daa

Search #2 Search terms for hepatitis C (n= 105 429)

“Hepatitis c” OR HCV OR “chronic hepatitis C” OR “Acute Hepatitis C”

Search #3 Search terms for Ribavirin (n= 18 549)

Ribavirin OR Rebetol OR Ribasphere OR RibaPak OR Copegus OR Virazole OR Moderiba OR "Tribavirin" OR "Vilona" OR "Viramide" OR "Virazide" OR “Virazid” OR "ICN-1229" OR "ICN 1229" OR "ICN1229" OR "Ribamide" OR "Ribamidil" OR "Ribamidyl" OR "RBV"

Search #4 - #1 AND #2 AND #3 (n= 3 014)

Search #5 – Search #4 filter from 2010 – 2022 (n= 3 000)

Cochrane Central Register of Controlled Trials (CENTRAL) search

Search #1 – (n=11 999)
sofosbuvir OR sovaldi OR simeprevir OR olysio OR daclatasvir OR daklinza OR ledipasvir OR harvoni OR elbasvir OR grazoprevir OR zepatier OR velpatasvir OR epclusa OR ombitasvir OR paritaprevir OR dasabuvir OR viekira pak OR voxilaprevir OR ritonavir OR 3D OR glecaprevir OR pibrentasvir OR mavyret OR direct-acting agents OR direct acting antiviral OR daa

Search #2 – (n= 10 078)
"Hepatitis c" OR HCV OR "chronic hepatitis C" OR "Acute Hepatitis C"

Search #3- (n= 4 802)
Ribavirin OR Rebetol OR Ribasphere OR RibaPak OR Copegus OR Virazole OR Moderiba OR "Tribavirin" OR "Vilona" OR "Viramide" OR "Virazide" OR “Virazid” OR "ICN-1229" OR "ICN 1229" OR "ICN1229" OR "Ribamide" OR "Ribamidil" OR "Ribamidyl" OR "RBV"

Search#4- #1 AND #2 AND #3 (n= 1 235)

Search #5 – Search #4 filter from 2010 – 2022 (n= 1 211)

SCOPUS search

Search #1 – (n= 971 103)

"sofosbuvir" OR "sovaldi" OR "simeprevir" OR "olysio" OR "daclatasvir" OR "daklinza" OR "ledipasvir" OR "harvoni" OR "elbasvir" OR "grazoprevir" OR "zepatier" OR "velpatasvir" OR "epclusa" OR "ombitasvir" OR "paritaprevir" OR "dasabuvir" OR "viekira pak" OR voxilaprevir OR ritonavir OR 3d OR glecaprevir OR pibrentasvir OR mavyret OR "direct-acting agents" OR "direct acting antiviral" OR daa

Search #2- (n= 384 135)

"Hepatitis c" OR HCV OR "chronic hepatitis C" OR "Acute Hepatitis C"

Search #3- (n= 78 160)

Ribavirin OR Rebetol OR Ribasphere OR RibaPak OR Copegus OR Virazole OR Moderiba OR "Tribavirin" OR "Vilona" OR "Viramide" OR "Virazide" OR “Virazid” OR "ICN-1229" OR "ICN 1229" OR "ICN1229" OR "Ribamide" OR "Ribamidil" OR "Ribamidyl" OR "RBV"

Search #4 - #1 AND #2 AND #3 (n= 16 141)

Search #5 – Search #4 filter from 2010 – 2022 (n= 15 405)

Cumulative Index to Nursing and Allied Health Literature (CINAHL)

Search #1 – (n= 54 788)

"sofosbuvir" OR "sovaldi" OR "simeprevir" OR "olysio" OR "daclatasvir" OR "daklinza" OR "ledipasvir" OR "harvoni" OR "elbasvir" OR "grazoprevir" OR "zepatier" OR "velpatasvir" OR "epclusa" OR "ombitasvir" OR "paritaprevir" OR "dasabuvir" OR "viekira pak" OR voxilaprevir OR ritonavir OR 3d OR glecaprevir OR pibrentasvir OR mavyret OR "direct-acting agents" OR "direct acting antiviral" OR daa

Search #2- (n= 21 706)

"Hepatitis c" OR HCV OR "chronic hepatitis C" OR "Acute Hepatitis C"

Search #3- (n= 3 164)

Ribavirin OR Rebetol OR Ribasphere OR RibaPak OR Copegus OR Virazole OR Moderiba OR "Tribavirin" OR "Vilona" OR "Viramide" OR "Virazide" OR “Virazid” OR "ICN-1229" OR "ICN 1229" OR "ICN1229" OR "Ribamide" OR "Ribamidil" OR "Ribamidyl" OR "RBV"

Search #4 - #1 AND #2 AND #3 (n= 617)

Search #5 – Search #4 filter from 2010 – 2022 (n= 616)

MedRxif- the preprint server for Health Sciences

Search #1 – (n= 26 350)

"direct-acting agents" OR "direct acting antiviral" OR daa OR "sofosbuvir" OR "sovaldi"

Search #2- (n= 5)

We manually selected relevant articles from the first 1000 results.

Updated search conducted on 17th of September 2023

PubMed search

Search #1 Search terms for DAAs (n= 309 321)

"sofosbuvir" OR "sovaldi" OR "simeprevir" OR "olysio" OR "daclatasvir" OR "daklinza" OR "ledipasvir" OR "harvoni" OR "elbasvir" OR "grazoprevir" OR "zepatier" OR "velpatasvir" OR "epclusa" OR "ombitasvir" OR "paritaprevir" OR "dasabuvir" OR "viekira pak" OR voxilaprevir OR ritonavir OR 3D OR glecaprevir OR pibrentasvir OR mavyret OR "direct-acting agents" OR “direct acting antiviral” OR daa

Search #2 Search terms for hepatitis C (n= 108 017)

“Hepatitis c” OR HCV OR “chronic hepatitis C” OR “Acute Hepatitis C"

Search #3 Search terms for Ribavirin (n= 18 817)

Ribavirin OR Rebetol OR Ribasphere OR RibaPak OR Copegus OR Virazole OR Moderiba OR "Tribavirin" OR "Vilona" OR "Viramide" OR "Virazide" OR “Virazid” OR "ICN-1229" OR "ICN 1229" OR "ICN1229" OR "Ribamide" OR "Ribamidil" OR "Ribamidyl" OR "RBV"

Search #4 - #1 AND #2 AND #3 (n= 3 069)

Search #5 – Search #4 filter from 17th November 2022 to 17th September 2023 (n=65)

SCOPUS search

Search #1 – (n= 1 060 801)

"sofosbuvir" OR "sovaldi" OR "simeprevir" OR "olysio" OR "daclatasvir" OR "daklinza" OR "ledipasvir" OR "harvoni" OR "elbasvir" OR "grazoprevir" OR "zepatier" OR "velpatasvir" OR "epclusa" OR "ombitasvir" OR "paritaprevir" OR "dasabuvir" OR "viekira pak" OR voxilaprevir OR ritonavir OR 3d OR glecaprevir OR pibrentasvir OR mavyret OR "direct-acting agents" OR "direct acting antiviral" OR daa

Search #2- (n= 150 500)

"Hepatitis c" OR HCV OR "chronic hepatitis C" OR "Acute Hepatitis C"

Search #3- (n= 40 077)

Ribavirin OR Rebetol OR Ribasphere OR RibaPak OR Copegus OR Virazole OR Moderiba OR "Tribavirin" OR "Vilona" OR "Viramide" OR "Virazide" OR “Virazid” OR "ICN-1229" OR "ICN 1229" OR "ICN1229" OR "Ribamide" OR "Ribamidil" OR "Ribamidyl" OR "RBV"

Search #4 - #1 AND #2 AND #3 (n= 6 760)

Search #5 – Search #4 filter from 2022 – 2023 (n= 469)

Cumulative Index to Nursing and Allied Health Literature (CINAHL)

Search #1 – (n= 57 312)

"sofosbuvir" OR "sovaldi" OR "simeprevir" OR "olysio" OR "daclatasvir" OR "daklinza" OR "ledipasvir" OR "harvoni" OR "elbasvir" OR "grazoprevir" OR "zepatier" OR "velpatasvir" OR "epclusa" OR "ombitasvir" OR "paritaprevir" OR "dasabuvir" OR "viekira pak" OR voxilaprevir OR ritonavir OR 3d OR glecaprevir OR pibrentasvir OR mavyret OR "direct-acting agents" OR "direct acting antiviral" OR daa

Search #2- (n= 21 960)

"Hepatitis c" OR HCV OR "chronic hepatitis C" OR "Acute Hepatitis C"

Search #3- (n= 3 115)

Ribavirin OR Rebetol OR Ribasphere OR RibaPak OR Copegus OR Virazole OR Moderiba OR "Tribavirin" OR "Vilona" OR "Viramide" OR "Virazide" OR “Virazid” OR "ICN-1229" OR "ICN 1229" OR "ICN1229" OR "Ribamide" OR "Ribamidil" OR "Ribamidyl" OR "RBV"

Search #4 - #1 AND #2 AND #3 (n= 611)

Search #5 – Search #4 filter from 2022 – 2023 (n= 20)
