# Supplementary material for: Efficacy and Safety of Adding Ribavirin to Sofosbuvir-Based Direct-Acting Antivirals (DAAs) in Re-Treating Non-Genotype 1 Hepatitis C—A Systematic Review and Meta-Analysis
Source: Diseases. 2025 Apr 29;13(5):138. doi: 10.3390/diseases13050138 (PMC12110649; doi:10.3390/diseases13050138)
Supplement: Supplementary file 1 [file diseases-13-00138-s001.zip › Supplementary graphs and tables.pdf]

Supplementary Figure S1- Quality assessment of included studies

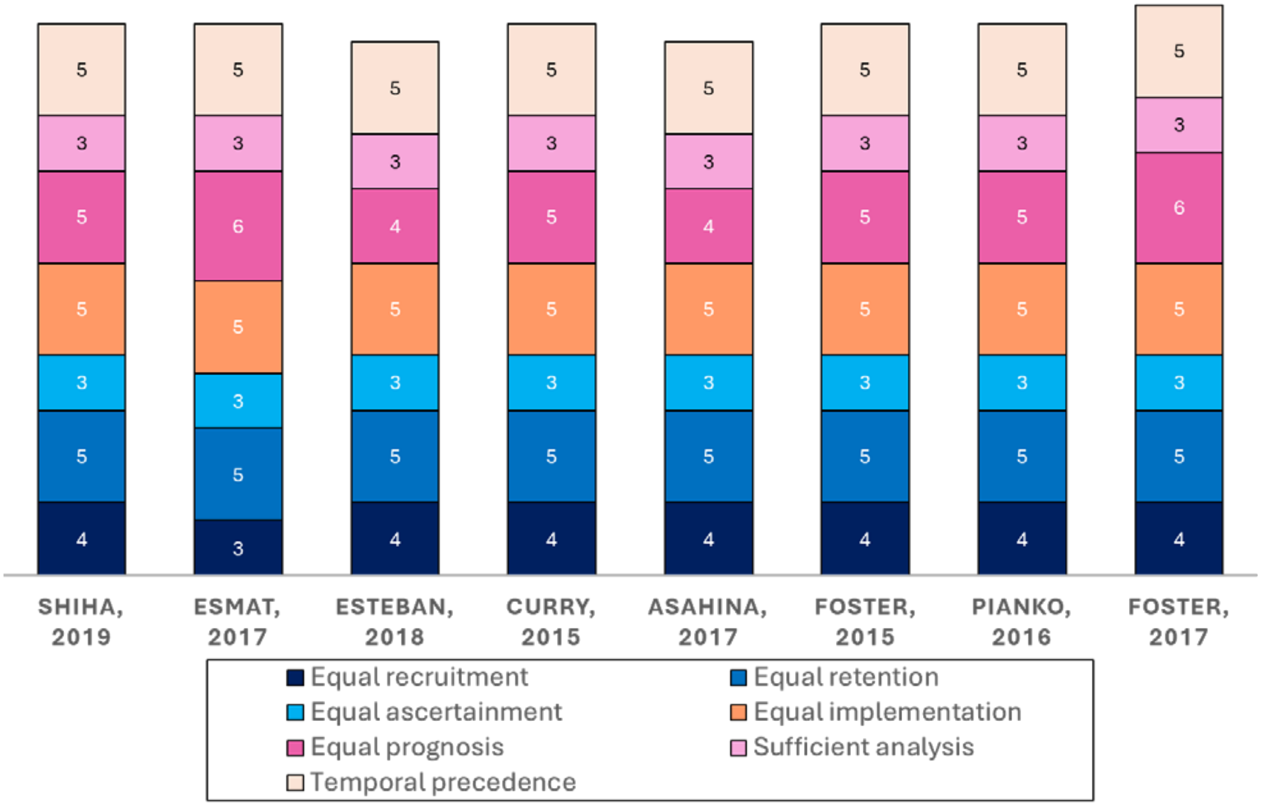

NB: *Equal Recruitment* **(navy)** has 4 safeguard**s,** *Equal Retention* **(dark blue)** has 5 safeguards**,** *Equal Ascertainment* **(blue)** has 7 safeguards**,** *Equal Implantation* **(orange)** has 6 safeguards**,** *Equal Prognosis* **(pink)** has 6 safeguards**,** *Sufficient Analysis* **(light pink)** has 3 safeguards**,** *Temporal Precedence* has 5 safeguards **(beige).**

Supplementary Figure S2- Assessment of heterogeneity for the main outcome

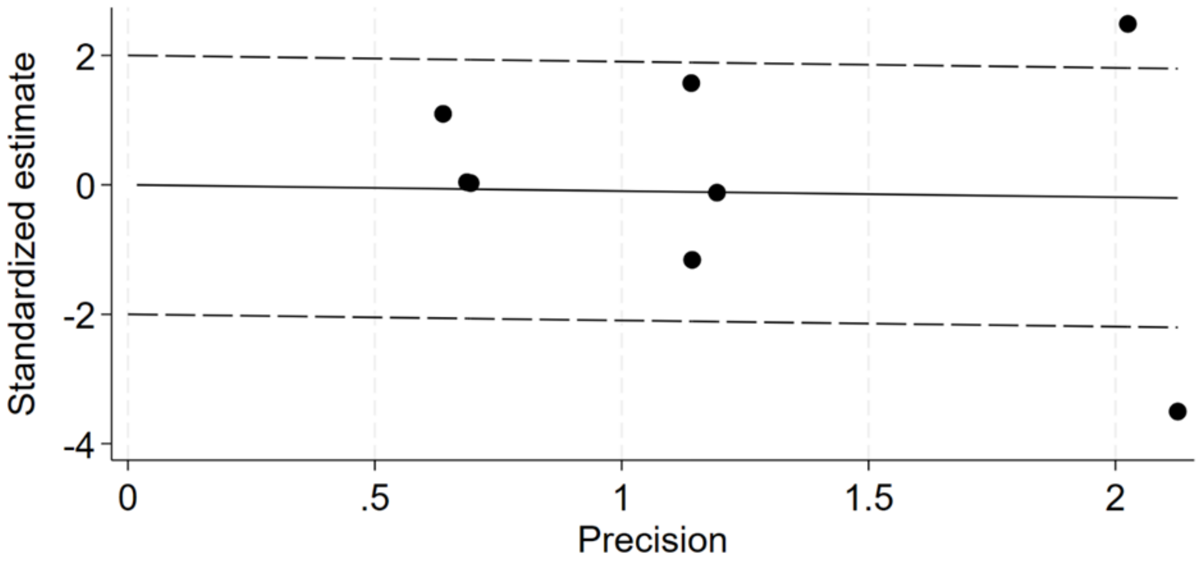

The Galbraith plot shows all the studies that reported SVR at 12 weeks in treatment-experienced non-genotype 1 participants. The regression line is almost horizontal showing an effect near the null. All studies, except two, fall within the 95% CIs (the dashed lines) suggesting little heterogeneity.

Supplementary Figure S3- Doi and funnel plots for SVR12

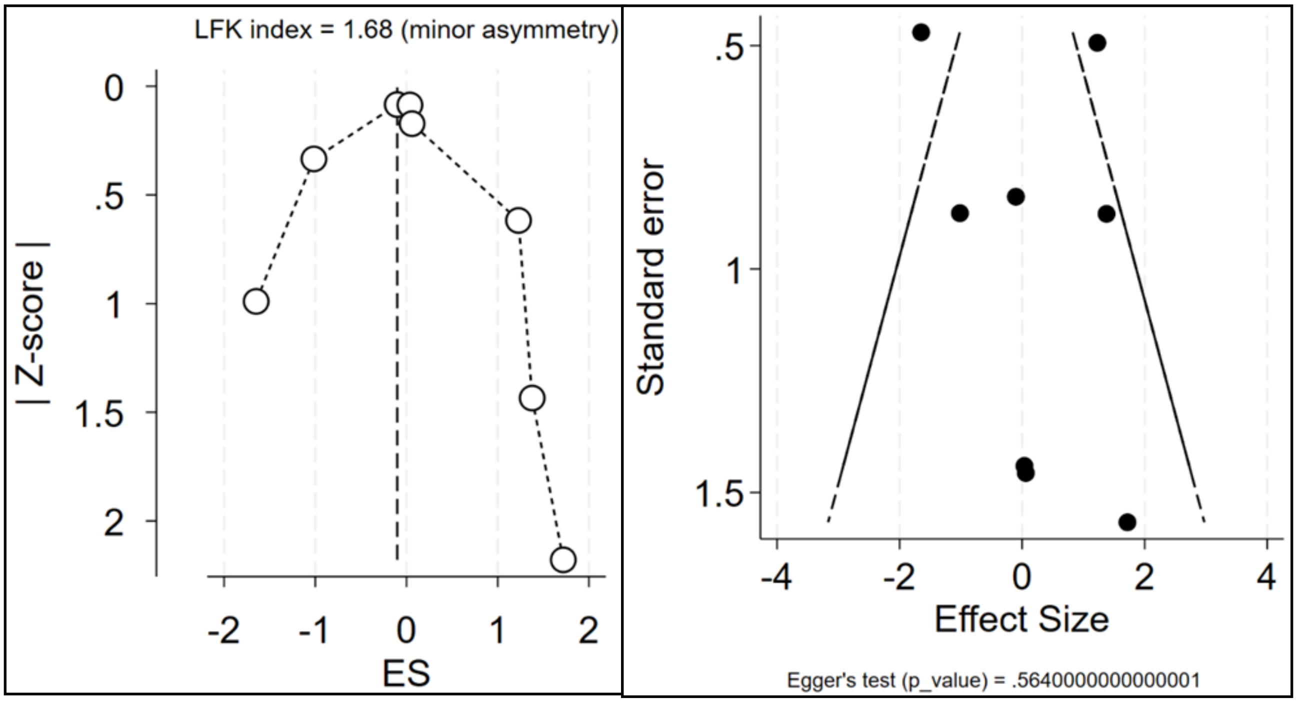

In the Doi plot, the studies form the two limbs of the funnel, if the limbs of the funnel show unequal divergence from the center of the funnel or one limb has more studies in comparison to the other this shows asymmetry or the presence of publication bias. In this Doi plot, there is minor asymmetry, confirmed by the LFK index of 1.68. The funnel plot shows the relation between the study's effect size (OR) and its precision. On visual inspection of the funnel plot, the included studies are roughly symmetrical distributed either side of the funnel, indicating symmetry and that publication bias is less likely. Egger's test p-value was 0.564 indicating weak evidence against the null hypothesis of symmetry.

Supplementary Figure S4- Sensitivity analysis

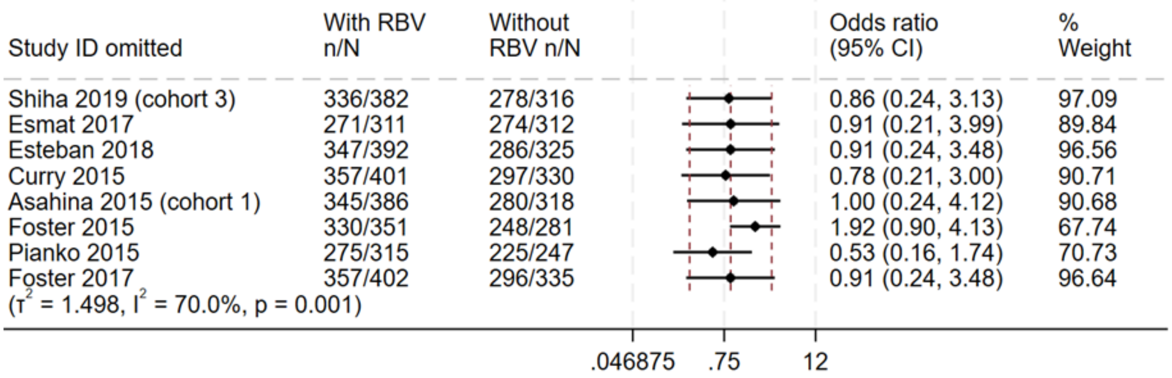

These forest plot show the sensitivity analysis by the leave-one-out analysis, for the main outcome of SVR 12 to examine the influence of each study on the meta-analytic outcome estimate. The results support those of the main analysis of SVR 12 that ribavirin does not improve SVR 12 in treatment-experienced non-genotype 1 HCV patients.

Supplementary Table S1- Subgroup analysis for SVR at 12 weeks

Subgroups analysis				
**Groups**	**SVR at 12 weeks, OR (95%CI)**	**Number of studies (participants)**	**I^2**	**LFK index (for publication bias)**
Genotype 3	0.78 (0.09-6.63)	4 (458)	83.2%	0.04 (no asymmetry)
Genotype 4	1.39 (0.31-6.31)	2 (223)	4.7%	3.03 (major asymmetry)

This table shows the results of subrgoup analyses, including the odds ratio for the primary outcome of SVR at 12 weeks, the number of studies and participants in each subgroup, the I^2 for heterogeneity, and the LFK index. The risk ratio remained almost constant, suggesting robustness of the meta-analytic results.

Abbreviations: *Confidence Interval (CI), Heterogeneity (I^2), Odds Ratio (OR)*

Supplementary Figure S5- Subgroup analysis by cirrhosis status

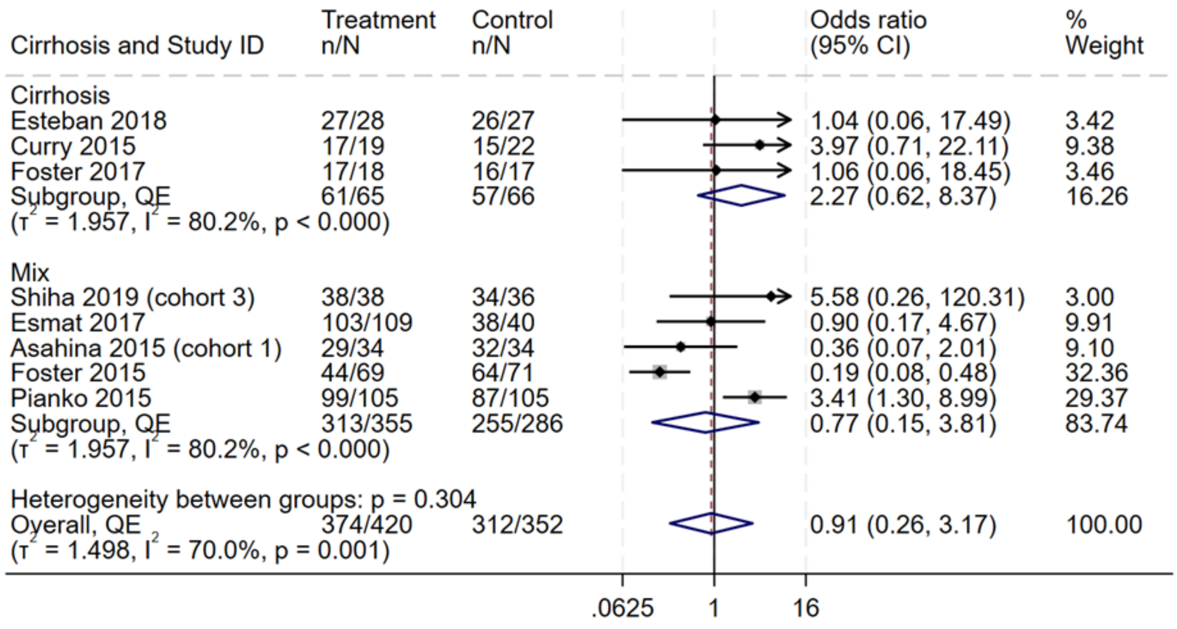

This forest plot shows that there is no additional odds of achieving SVR12 when adding ribavirin to sofosbuvir-based regimens compared to sofosbuvir-based regimens alone in participants with cirrhosis and those with or without cirrhosis (OR 2.27) and (OR 0.77), respectively. Additionally, the I^2 for heterogeneity decreases slightly, suggesting that some of the heterogeneity between the study effect measures could be explained by the cirrhosis status of the individual.

Supplementary Figure S6- Heterogeneity assessment for the analysis of any adverse events

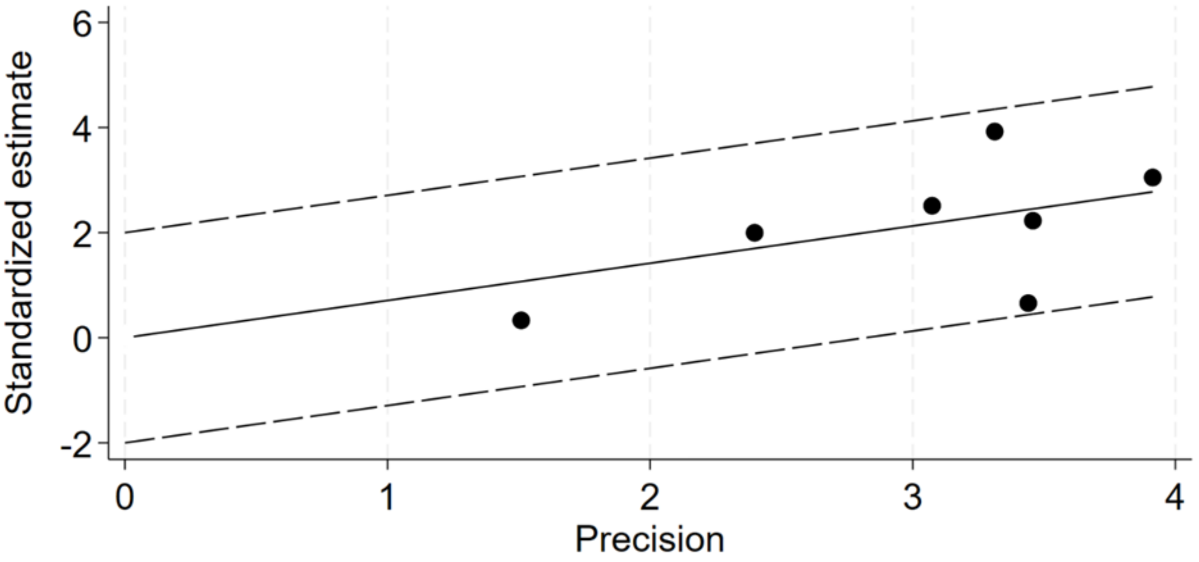

The Galbraith plot shows the seven studies that reported the development of any adverse events in individuals with non-genotype 1 HCV. The regression line is sloping upward, meaning that the odds ratio is more than 1, suggesting an increase in the odds of developing adverse events when ribavirin was added to sofosbuvir-based regimens as compared to sofosbuvir-based regimens alone. All studies fall within the 95% CIs (the dashed lines) suggesting no heterogeneity.

Supplementary Figure S7- Publication bias assessment for the analysis of any adverse events

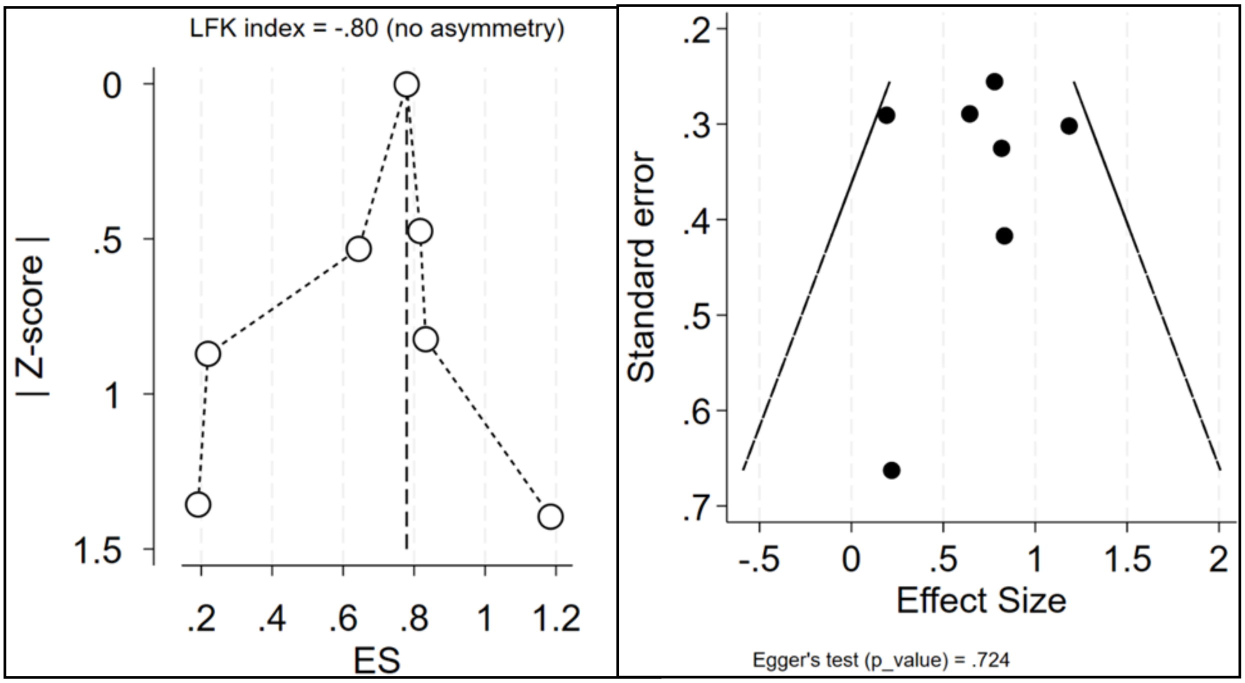

The Doi plot shows no asymmetry, confirmed by the LFK index = -0.8. Visual inspection of the funnel plot shows some symmetry of the distribution of the included studies inside the funnel, confirmed by the Egger's test = 0.724 suggesting that there is weak evidence against the null hypothesis of symmetry.

Supplementary Figure S8- Forest plot for treatment discontinuation analysis

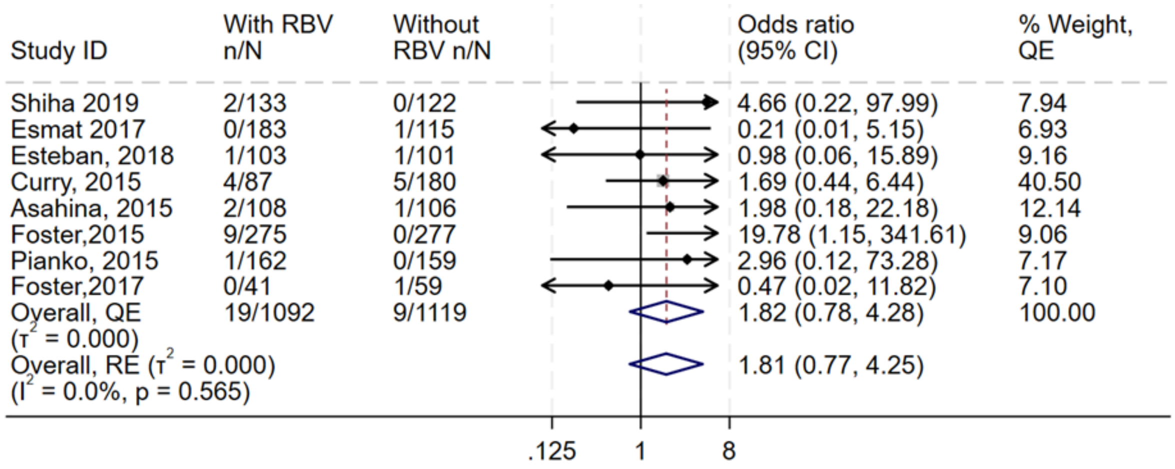

This forest plot shows the overall analysis for the odds of treatment discontinuation in the treatment group (sofosbuvir-based combinations and ribavirin) compared to the control group (DAAs only) which was reported by all eight studies. The meta-analytic effect size using the Quality effects and Random effects models are (OR 1.82), (OR 1.81), respectively.

Supplementary Figure S9- Heterogeneity assessment for the analysis of treatment discontinuation

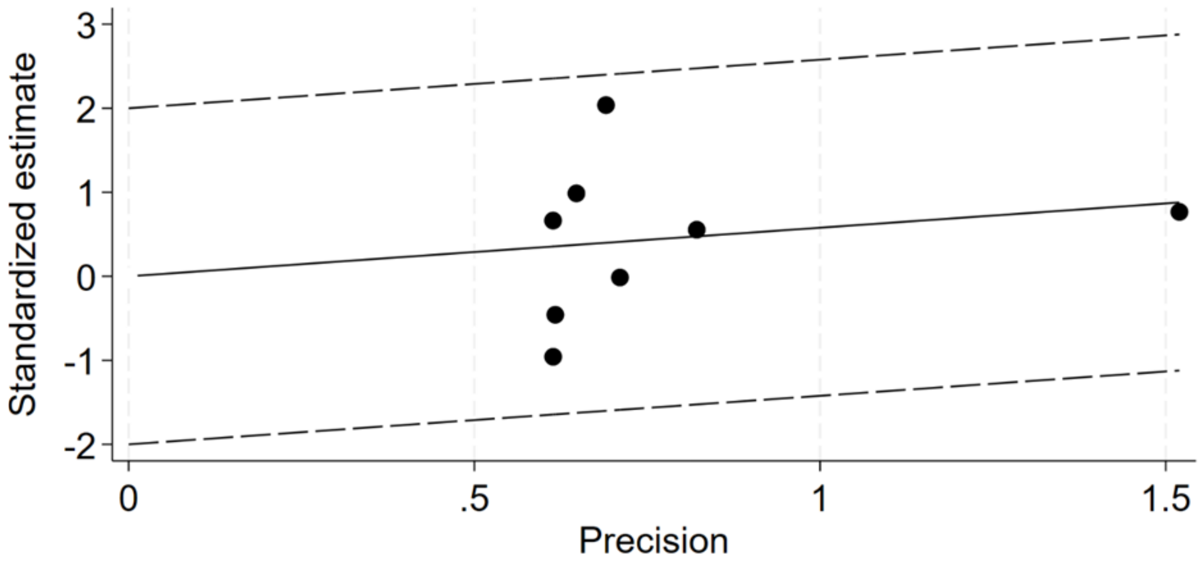

The Galbraith plot shows the eight studies that reported drug discontinuation in participants with non-genotype 1 HCV. The regression line is sloping upward, meaning that the risk ratio is more than 1, suggesting an increase in the odds of discontinuing treatment when ribavirin was added to sofosbuvir-based regimens as compared to DAAs alone. All studies fall within the 95% CIs (the dashed lines) suggesting no heterogeneity.

Supplementary Figure S10- Publication bias assessment for the analysis of treatment discontinuation

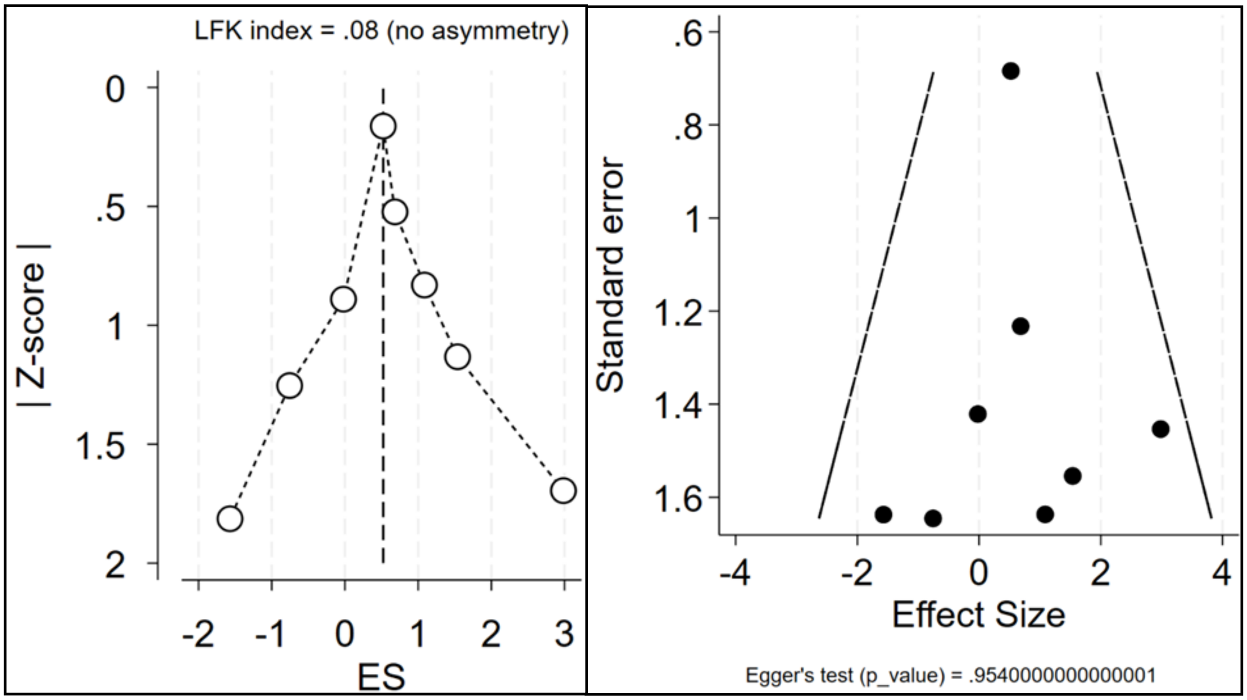

The Doi plot shows no asymmetry, confirmed by the LFK index = 0.08. Visual inspection of the funnel plot shows symmetry of the distribution of the included studies inside the funnel, confirmed by the Egger's test = 0.954 suggesting that there is low evidence against the null hypothesis of symmetry.

Supplementary Figure S11- Forest plot for serious adverse events analysis

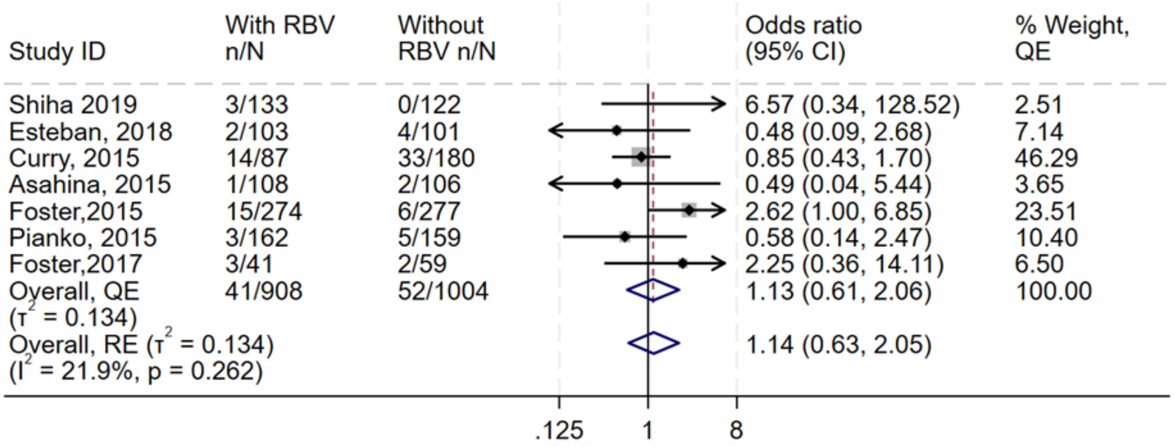

This forest plot shows the analysis for the odds of developing serious adverse events in the treatment group (DAAs and ribavirin) compared to the control group (DAAs only) which was reported by seven studies. The meta-analytic effect size using the Quality effects and Random effects models are (RR 1.13) and (RR 1.14), respectively.

Supplementary Figure S12- Heterogeneity assessment for the analysis of developing serious adverse events

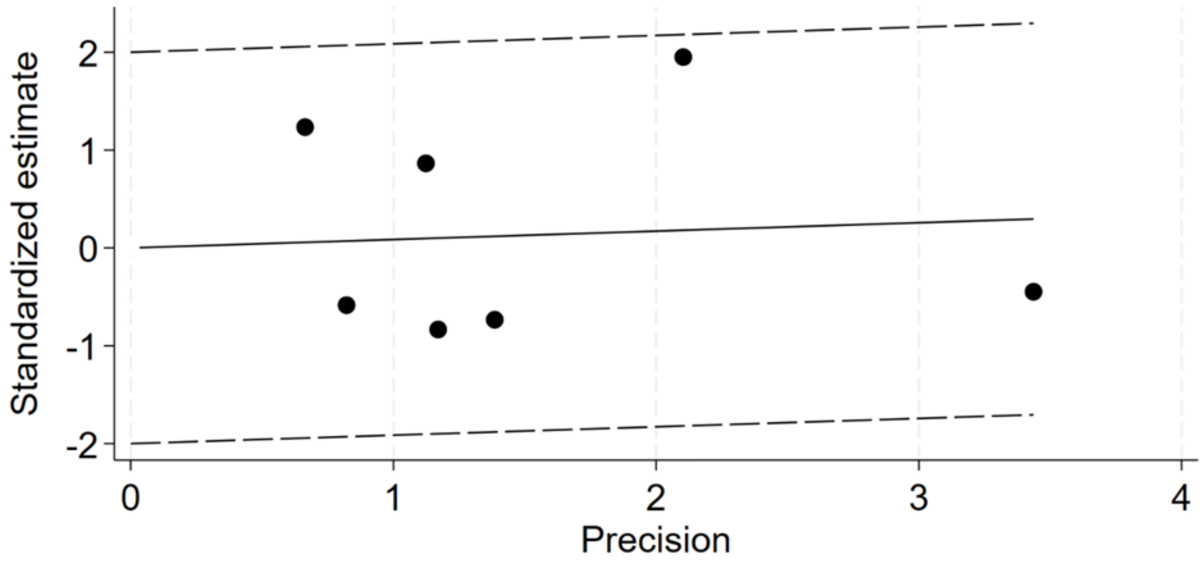

The Galbraith plot shows the seven studies that reported the development of any adverse events in non-genotype 1 participants. The regression line is almost horizontal, showing an effect near the null, suggesting no increase in the risk of developing serious adverse events when ribavirin was added to DAAs as compared to DAAs alone. All studies fall within the 95% CIs (the dashed lines) suggesting no heterogeneity.

Supplementary Figure S13- Publication bias assessment for the analysis of developing serious adverse events

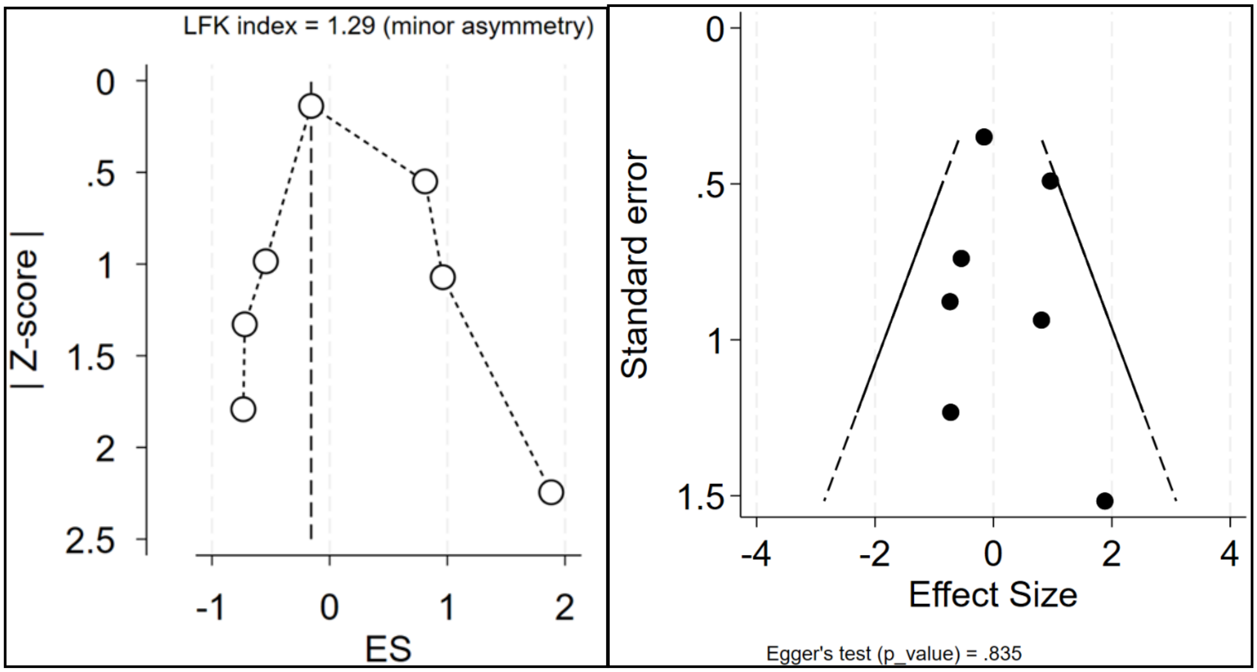

The *Doi plot shows minor asymmetry, confirmed by the LFK index = 1.29. Visual inspection of the funnel plot shows symmetry of the distribution of the included studies inside the funnel, confirmed by the Egger's test = 0.835 suggesting that there is low evidence against the null hypothesis of symmetry.*
